# Identification and Characterization of Amlexanox as a G Protein-Coupled Receptor Kinase 5 Inhibitor

**DOI:** 10.3390/molecules191016937

**Published:** 2014-10-22

**Authors:** Kristoff T. Homan, Emily Wu, Alessandro Cannavo, Walter J. Koch, John J. G. Tesmer

**Affiliations:** 1 Life Sciences Institute and the Departments of Pharmacology and Biological Sciences, University of Michigan, Ann Arbor, MI 48109, USA; 2 Center for Translation Medicine, Temple University School of Medicine, Philadelphia, PA 19140, USA

**Keywords:** drug screening, differential scanning fluorometry, inhibitor, G protein-coupled receptor kinase, structure, amlexanox

## Abstract

G protein-coupled receptor kinases (GRKs) have been implicated in human diseases ranging from heart failure to diabetes. Previous studies have identified several compounds that selectively inhibit GRK2, such as paroxetine and balanol. Far fewer selective inhibitors have been reported for GRK5, a target for the treatment of cardiac hypertrophy, and the mechanism of action of reported compounds is unknown. To identify novel scaffolds that selectively inhibit GRK5, a differential scanning fluorometry screen was used to probe a library of 4480 compounds. The best hit was amlexanox, an FDA-approved anti-inflammatory, anti-allergic immunomodulator. The crystal structure of amlexanox in complex with GRK1 demonstrates that its tricyclic aromatic ring system forms ATP-like interactions with the hinge of the kinase domain, which is likely similar to how this drug binds to IκB kinase ε (IKKε), another kinase known to be inhibited by this compound. Amlexanox was also able to inhibit myocyte enhancer factor 2 transcriptional activity in neonatal rat ventricular myocytes in a manner consistent with GRK5 inhibition. The GRK1 amlexanox structure thus serves as a springboard for the rational design of inhibitors with improved potency and selectivity for GRK5 and IKKε.

## 1. Introduction

The identification of compounds that modulate the activity of G protein-coupled receptors (GPCRs) has proven to be fertile ground for the development of clinically relevant therapeutics. However, there has recently been a move to identify compounds with more subtle effects on GPCR signaling, such as those capable of inducing biased signaling [1] or of inhibiting the function of regulatory proteins that act on GPCRs, such as the G protein-coupled receptor kinases (GRKs). GRKs chiefly function to initiate the deactivation of agonist occupied GPCRs primarily through phosphorylation of their cytoplasmic C-terminal tails, which leads to the recruitment of arrestins and ultimately clathrin-mediated endocytosis [2]. Individual GRKs are validated targets for the treatment of human conditions ranging from heart failure [3,4] to diabetes [5,6]. Although GRK2 has thus far been the primary focus of drug discovery efforts [7,8,9,10,11], GRK5 has been increasingly targeted as a potential treatment for cardiac hypertrophy [12,13]. The development of inhibitors selective for GRK5 over GRK2 has been challenging due to high sequence identity in their active sites. However, some compounds that are relatively selective for GRK5 have now been reported [9,14], but there is as of yet no crystal structure of any these molecules in complex with GRK5, and thus their mechanisms dictating selectivity are ill-defined. To identify novel scaffolds that selectively inhibit GRK5, multiplexed differential scanning fluorometry (DSF) was to screen 4480 compounds in the Center of Chemical Genomics at the University of Michigan, leading to the identification of the FDA-approved small molecule amlexanox as a weakly selective GRK5 inhibitor. The crystal structure of amlexanox in complex with GRK1, a closely related GRK with 55% sequence identity to GRK5 in its kinase domain, was solved, providing molecular insights into how this compound interacts with GRKs and likely other protein kinases targeted by this drug such as IκB kinase ε (IKKε) [15].

## 2. Results and Discussion

### 2.1. High-Throughput Screening

**Figure 1:**
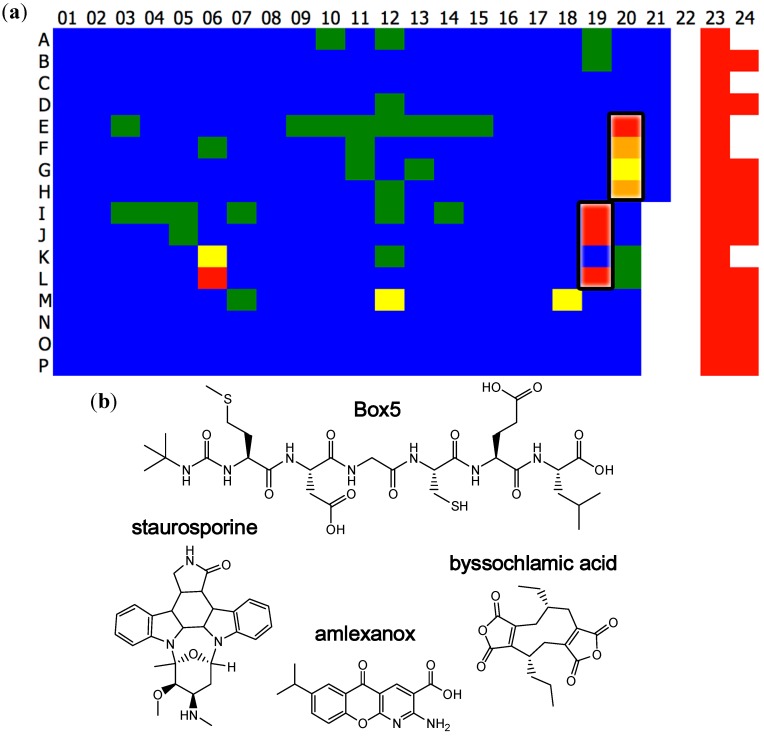
Differential scanning fluorometry (DSF) screen for compounds that alter the melting point of GRK5. (**a**) Representative heat map of a 384-well plate containing individual compounds from the deconvolution step of the screen. Colors correspond to >80% (red), 61%–80% (orange), 41%–60% (yellow), 21%–40% (green), <20% (blue) of a 3 standard deviation shift in the GRK5 melting point relative to the negative controls. Negative buffer-only controls occupy columns 1 and 2, non-protein containing controls (white) and positive ADP controls occupy columns 21 through 24. Individual compounds were evaluated 4 times in adjacent wells (running along columns). Compounds carried forward in the analysis were required to produce > 3σ shift in the GRK5 melting point in at least 3 of the 4 wells. Staurosporine and Box5 are highlighted in wells 19 I–L and 20 E–G, respectively. (**b**) Chemical structures of the four hits identified in the screen.

DSF was conducted in order to rapidly and inexpensively explore a multiplexed small molecule library for molecules that can alter the melting point of bovine GRK5. Screening via a tagless platform such as DSF eliminates the need for labeled secondary interaction partners such as the RNA aptamer used in previous screens to identify inhibitors of GRK2 [11], although a caveat is that not all compounds that bind will necessarily inhibit activity. A collection of 4480 molecules at the Center for Chemical Genomics at the University of Michigan (including MicroSource Spectrum and elements of the ChemDiv collection) was 4-fold multiplexed (4 compounds/well) and screened for small molecules capable of perturbing the melting point of GRK5. In the initial set of deconvolution measurements, there were 60 wells (240 compounds) chosen for follow-up analysis that altered the melting point of GRK5 by at least three standard deviations for positive (>1.7 °C) or eight standard deviations for negative (<−4.9 °C) shifts compared to the negative controls. The greater stringency imposed for negative shifts reflected the greater number of observed large negative shifts. These 240 compounds were assayed individually in quadruplicate with compounds at a final concentration of 25 μM (Figure 1a). Of these compounds, 20 altered the melting temperature greater than three standard deviations as compared with buffer only controls and were chosen for dose response analysis, after which only 4 compounds were left that caused melting point shifts of 3.0–4.9 standard deviations (Figure 1b). No destabilizing compounds were confirmed as hits. One compound, byssochlamic acid, was not commercially available and could not be subjected to further analysis. The other three compounds (staurosporine, a promiscuous kinase inhibitor [16], a WNT5a antagonist (Box5) [17], and amlexanox, an IKKε/Tank-binding kinase (TBK) inhibitor [15]) were reconfirmed by replating library compounds and repeating the DSF measurements. Staurosporine, which served effectively as a positive control, was not analyzed further. Fresh powder stocks of Box5 and amlexanox were purchased and subjected to validation in secondary assays.

### 2.2. Biochemical Validation

**Figure 2:**
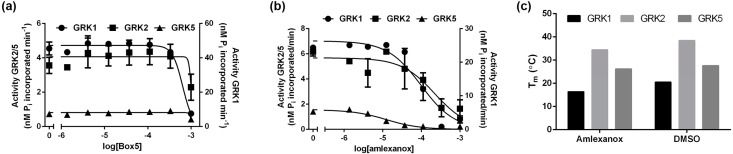
Amlexanox inhibits GRK activity. (**a**) Box5 only inhibits at high concentrations in a manner consistent with nonspecific inhibition. Shown are representative dose response curves performed in duplicate representing inhibition of rhodopsin phosphorylation by GRK1 (circles), GRK2 (squares), and GRK5 (triangles). (**b**) Amlexanox inhibits rhodopsin phosphorylation by GRK5 with higher potency than GRK1 and GRK2. In panels a and b, error bars represent the standard error of the mean (SEM). (**c**) Amlexanox (200 µM) reduces the melting temperature of GRK1, GRK2, and GRK5 by 4.1, 4.0, and 1.4 °C, respectively, relative to DMSO controls. Thus, amlexanox destabilizes GRKs at this concentration. Data shown is representative of 3 experiments performed in triplicate, error bars represent the SEM.

The two remaining lead compounds were evaluated as inhibitors of GRK-mediated phosphorylation of rhodopsin in rod outer segments [10,11]. Bovine GRK1 and GRK2 were used as representatives of the GRK1 and GRK2 sub-families of GRKs to assess selectivity. The WNT5a peptide inhibitor (Box5) only showed inhibition of GRKs at concentrations greater than 500 μM and exhibited a steep Hill coefficient (Figure 2a), consistent with the formation of aggregates [18,19], and was not further considered. In contrast, amlexanox exhibited micromolar potency of inhibition against GRK5 (plogIC_50_ = 4.9) (Figure 2b), and inhibited GRK1 and GRK2 less potently (plogIC_50_ = 4.2 and 3.9, respectively). Thus, amlexanox inhibits GRK5 with 5- to 10-fold selectivity over the other GRKs. When the melting point for each GRK was determined in the presence of purchased amlexanox at 200 µM, GRK5 was the least altered (ΔT_m_ = −1.4 °C) as compared with GRK1 (ΔT_m_ = −4.1 °C) and GRK2 (ΔT_m_ = −4.0 °C) (Figure 2c). This is in contrast to the positive shift in melting point observed for GRK5 in the primary and deconvolution screens. This is a concentration dependent effect, as amlexanox at final concentrations of 10 , 67, 125, 200, and 250 µM caused shifts in the melting point of GRK5 by 1.0, 1.2, 0.8, −1.4 and −2.0 °C, respectively (data not shown), suggesting that the drug may be destabilizing proteins nonspecifically when used in molar excess.

### 2.3. Cellular Activity

**Figure 3:**
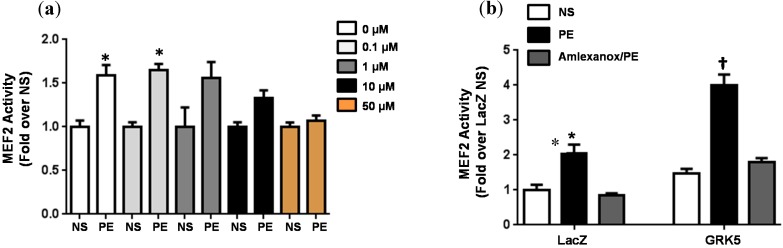
MEF2 activity in NRVMs measured using a luciferase assay system. (**a**) Cells were infected with an adenovirus expressing a MEF2-luciferase (Ad-MEF2-Luc) reporter construct for 48 h. Following infection, the cells were either not stimulated (NS), or stimulated for 24 h with phenylephrine (PE, 50 µM) or with both PE and amlexanox at different doses (0 µM, 0.1 µM, 1 µM, 10 µM and 50 µM). * *p* < 0.05 *vs.* NS, as assessed by one-way ANOVA with a Bonferroni correction. (**b**) Cells were co-infected with the Ad-MEF2-Luc and either Ad-LacZ or Ad-GRK5 and then incubated with or without 50 µM PE for 24 h or with both 50 µM amlexanox and PE. * *p* < 0.05 *vs*. LacZ NS, † *p* < 0.001 *vs.* all, as assessed by one-way ANOVA with a Bonferroni correction.

GRK5 phosphorylates histone deacetylase 5 (HDAC5), which promotes translocation of the protein out of the nucleus of cardiomyocytes [20]. Class II histone deacetylase proteins such as HDAC5 are transcriptional repressors of myocyte enhancer factor 2 (MEF2) [21]. Thus, GRK5 mediated phosphorylation of HDAC5 activates MEF2, which contributes to cardiac hypertrophy. To determine whether amlexanox is capable of inhibiting GRK5 *in cellulo*, phenylephrine-induced MEF2 activation in neonatal rat ventricular myocytes (NRVMs) was monitored. Amlexanox was capable of blocking MEF2 activation at micromolar concentrations, with near abrogation of activation at 50 μM (Figure 3a). Furthermore, GRK5 overexpression, which caused greater MEF2 activation in these cells, was abolished upon administration of 50 μM amlexanox (Figure 3b).

### 2.4. Crystal Structure of the GRK1 Amlexanox Complex

**Figure 4:**
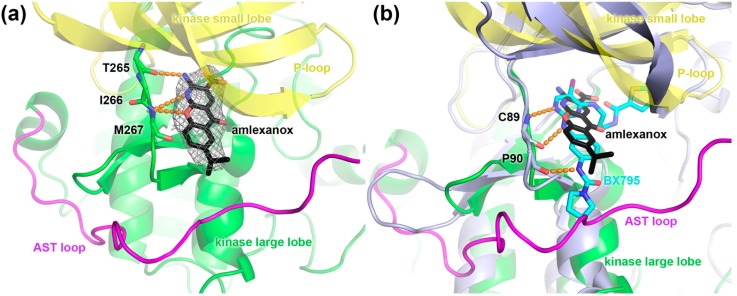
Crystal structure of GRK1 bound to amlexanox. (**a**) Amlexanox (stick model with black carbons) forms several hydrogen bonds (orange dashed lines) with the hinge of the GRK1 kinase domain (large lobe green and small lobe yellow). The isopropyl group of amlexanox is oriented to form hydrophobic interactions with the AST loop (magenta). Grey mesh corresponds to a 3σ |*F_o_*|-|*F_c_*| omit map. (**b**) Superposition of the kinase domain of GRK1 with that of TBK1 in complex with BX795 (cyan carbons) (PDB entry 4EUT, light blue) suggest that modifications to the amlexanox scaffold that fill the ribose and polyphosphate subsites of the active site, such as the thiophene arm of BX795, or that optimize contacts with the AST loop are routes to increase potency and/or selectivity.

In order to determine how amlexanox interacts with GRKs, the atomic structure of GRK1 in complex with the drug was determined at 2.82 Å resolution (Table 1). GRK1 was used as a surrogate for GRK5 because the structure of GRK5 has not yet been reported, GRK1 is known to readily crystallize in various ligand states [9], and GRK1 is a relatively close homolog of GRK5 with 47% sequence identity. The GRK1·amlexanox crystal structure was solved to 2.8 Å spacings and has four similar but non-identical complexes in the asymmetric unit. The largest conformational variation observed among them occurs in the active site tether (AST) loop that passes over the active site, which is disordered in one chain. Amlexanox induces a conformation in GRK1 very similar to that induced by ADP (PDB entry 3C4Z), leading to an overall RMSD of 1.4 Å for all 478 atomic pairs and requiring only a 0.3 Å translation of the large lobe relative to the small lobe to achieve the same conformation as calculated by DynDom [22,23]. Amlexanox exhibits strong omit map density in the active site of each monomer where its 2-aminopyridine group forms hydrogen bonds to backbone atoms of hinge residues Thr265, and Met267 (Figure 4a) in a manner similar to that observed in other reported GRK·inhibitor and adenine nucleotide complexes [9,10,11,24,25,26]. Its tricyclic ring system sandwiched between the side chains of Leu193, Val201, and Ala214 in the small lobe and the carbonyl of Met267 and the side chain of Leu321 in the large lobe. However, unlike previously reported GRK inhibitors, amlexanox does not form extensive interactions with the P-loop. Instead, the long axis of the drug extends out to form hydrophobic interactions with the AST loop in 3 of the 4 chains with its isopropyl group. This binding mode is similar to that of GSK2163632A in complex with GRK1 [9], wherein a large aromatic system of the compound packs primarily along the hinge and forms extensive interactions with the AST. Amlexanox is also a known inhibitor of IKKε and TBK1. The latter kinase has been crystallized in complex with a potent inhibitor (IC_50_ ~10 nM) known as BX795 (PDB entry 4EUT) [27]. Superposition of the kinase domains from the two structures (Figure 4b) illustrates that both inhibitors make multiple hydrogen bonds with the hinge of the kinase domain and pack such that the long axis of each compound extends towards the AST loop region of GRK1, although TBK1 lacks this element. Notably, BX795, which is orders of magnitude more potent than amlexanox, has an additional thiophene arm that extends under the P-loop of the active site such that it occupies the ribose and polyphosphate subsites, suggesting that these additional interactions are at least in part responsible for its higher potency *vs.* amlexanox.

**Table 1:** Crystallographic collection and refinement for the GRK1·amlexanox complex.

Protein Complex	GRK1·Amlexanox
X-ray source	APS 21-ID-E
Wavelength (Å)	0.9787
D_min_ (Å)	2.82 (2.87–2.82) *
Space group	*P* 2_1_ 2_1_ 2_1_
Cell constants (Å)	a = 118.1 b = 119.2 c = 174.3
Unique reflections	60016 (2932)
R_merge_ (%)	9.7% (100%)
Completeness (%)	100% (99.9%)
<I>/<σ_I_>	19.5 (1.4)
Redundancy	7.4 (7.1)
Refinement resolution (Å)	25–2.82 (2.88–2.82)
Total reflections used	56928 (3020)
RMSD bond lengths (Å)	0.005
RMSD bond angles (°)	0.919
Est. coordinate error (Å)	0.348
Ramachandran plot outliers (%)	3 (0.15%)
R_work_	24.1 (40.6)
R_free_	26.7 (44.4)
Protein atoms	15786
Water molecules	54
Inhibitor atoms	88
Average B-factor (Å^2^)	45.1
Protein	45.5
Inhibitor	36.2
MolProbity score	1.27
MolProbity Cβ deviations	0
MolProbity bad backbone bonds	0
MolProbity bad backbone angles	1
PDB Entry	4WBO

* Numbers in parentheses correspond to the highest resolution shell of data.

## 3. Experimental Section

### 3.1. Protein Purification

Rhodopsin in bovine rod outer segments (bROS) was prepared according to established protocols [28]. Bovine GRK1_1-535_, bovine GRK2_S670A_, bovine GRK5 was purified via sequential use of Ni-NTA metal affinity, Source15S cation exchange, and S200 size exclusion chromatography as previously described [10,11,29]*.* Proteins from bovine sources were used because they have >95% sequence identity with and do not exhibit any significant biochemical differences from their human homologs.

### 3.2. DSF Screen

Compounds dissolved in DMSO were four-fold multiplexed at 67 μM per compound (4 compounds/well) and loaded into black Thermo Fast 384 PCR plates leaving the first and last columns free for DMSO, no protein, and/or known compound controls (ADP). The primary screen was performed at 0.08 mg/mL GRK5 and 100 μM 1-anilinonaphthalene-8-sulfonic acid (ANS) in ThermoFluor buffer (20 mM HEPES pH 7.0, 5 mM MgCl_2_, 2 mM DTT, and 1 mM CHAPS). Wells that shifted the melting point of GRK5 higher by three standard deviations or lower than eight standard deviations compared to the DMSO controls (>1.7 °C or <−4.9 °C, respectively) were considered to be hits. These wells were deconvoluted and remeasured in quadruplicate in confirmatory experiments at 25 μM final ligand concentration. Compounds that exhibited shifts of greater than three standard deviations as compared with the buffer only controls in at least three of the four replicates in this step (20 molecules) were subjected to dose response analysis, of which four reconfirmed. Terminal confirmation DSF experiments were performed in ThermoFluor buffer with 0.2 mg/mL GRK5, 100 μM ANS, and represent triplicate measurements from at least three independent experiments.

### 3.3. Kinetic Assays

GRK kinetic assays were conducted in a buffer containing 20 mM HEPES pH 7.0, 2 mM MgCl_2_, and 0.025% n-dodecyl β-d-maltoside with 50 nM GRK and 500 nM ROS in 5 min reactions that were initiated with the addition of 5 μM ATP as previously reported [9,10,11]. Data was analyzed and inhibition curves were fit using GraphPad Prism. GRK5-mediated phosphorylation of the substrate tubulin has also been tested and yielded similar results: plogIC_50_ of 5.0 using tubulin as a substrate (data not shown) *versus* 4.9 using rhodopsin. Thus, there is no obvious substrate-dependent inhibition of GRK5 by amlexanox.

### 3.4. Cell Culture and Adenoviral Infection

NRVMs were isolated from 1- to 2-day old neonatal rat hearts as previously described [30]. NRVMs were cultured in DMEM supplemented with penicillin/streptomycin (100 U/mL) and 5% FBS at 37 °C in a 5% humidified atmosphere for 2–3 days. At 24 h post-isolation, NRVMs were infected with recombinant, replication-deficient adenoviruses expressing the following genes with their respective MOIs: GRK5 (50 MOI). Equal particles of an adenovirus expressing LacZ were used to control for non-specific adenoviral effects. Cells were cultured for 24 h prior to experimentation.

### 3.5. Luciferase Assay

NRVMs were co-infected with the Ad-MEF2-Luc and either the Ad-LacZ or Ad-GRK5 in the presence or absence of 50 µM PE for 24 h, or with both 50 µM amlexanox and PE. Cells were harvested in passive lysis buffer (Promega). Luciferase activity was measured according to manufacturer’s protocol (Promega) using a Victor plate reader [31].

### 3.6. GRK1·Paroxetine Crystal Structure Determination

Amlexanox (100 mM DMSO stock) and MgCl_2_ (500 mM stock) were added to a ~9 mg/mL bGRK1_535_ protein solution to attain a final concentration of 2 mM and 5 mM, respectively. Crystals were obtained via vapor diffusion using hanging drops consisting of 1.0 μL of protein and 1.0 μL of well solution containing 500 mM NaCl, 100 mM MES pH 6.25, and 20% PEG3350. Crystals appeared in approximately 1 week and continued to grow in size for at least one additional week. During harvesting, the crystals were cryoprotected by addition of 25% ethylene glycol to the drops prior to flash freezing in liquid nitrogen. Diffraction data were collected at the Advanced Photon Source (APS) on LS-CAT beamline ID-G at a wavelength of 0.9787 Å. Indexing, integration, and scaling were performed with HKL2000 [32]. A molecular replacement solution was achieved using the Phaser module of CCP4 and PDB entry 3C50 as the search model [33,34]. Refinement was performed with the Refmac5 module of CCP4 and model building was conducted with Coot [35,36]. The final model was validated with MolProbity [37] prior to deposition of coordinates and structure factors in the PDB as entry 4WBO.

## 4. Conclusions

This study identified amlexanox as a low micromolar inhibitor of GRK5 with modest selectivity over other GRK subfamilies, and demonstrated that the drug can significantly inhibit MEF2 transcriptional activity in NRVMs, consistent with inhibition of GRK5 in cells. The GRK1 amlexanox crystal structure demonstrates that the drug binds directly in the active site of the kinase in a manner that mimics the adenine ring of ATP. Amlexanox was also recently been identified as a similarly potent inhibitor of TBK1 and IKKε and was shown to exhibit anti-diabetic effects on mice fed high-fat diets [15]. Although a larger panel of kinases would have to be evaluated in order to know how promiscuous amlexanox is, its ability to inhibit diverse kinases likely reflects its structural similarity to adenine, in that in only forms hydrogen bonds to backbone atoms and that it occupies the same flat, aromatic binding pocket in the active site. There are no side chain substitutions in the GRK1 or GRK2 active sites that can readily explain their somewhat lower potency of inhibition relative to GRK5. Thus, differences in potency may simply reflect the ease by which each kinase can adopt a conformation conducive to the binding of amlexanox. A similar conclusion was reached when evaluating even more selective and potent inhibitors of GRK2 [25]. Regardless, the GRK1·amlexanox structure will serve as a useful platform to begin rational design of amlexanox-based therapeutics for the treatment of either cardiac hypertrophy (GRK5) or, by extension, diabetes (IKKε). Comparison of the GRK1·amlexanox and TBK1·BX795 crystal structures (Figure 4b) suggests two regions of the kinase active site could readily be exploited to increase selectivity and/or potency of future derivatives. First, optimizing interactions at the isopropyl end of amlexanox with the GRK5 AST loop, a region that has low sequence conservation with other GRKs and AGC kinases, should modulate the potency and selectivity of inhibition against GRK5 and other GRKs, but have little effect on TBK1/IKKε inhibition, as these kinases lack an AST element. Second, interactions with the P-loop introduced by modifications at the carboxyl end of the drug will likely afford higher affinity interactions, such as that exhibited by the thiophene arm of BX795. As the P-loop can exhibit dramatically different conformations in protein kinase crystal structures, even in those of the same protein kinase, these interactions may also lead to greater selectivity.
